# Evolution of BCL-2/IgH hybrid gene RNA expression during treatment of T(14;18)-bearing follicular lymphomas

**DOI:** 10.1038/sj.bjc.6690777

**Published:** 1999-11

**Authors:** P Soubeyran, I Hostein, M Debled, H Eghbali, I Soubeyran, F Bonichon, T Astier-Gin, B Hœrni

**Affiliations:** Departments of Molecular Oncology, Haematology, Pathology andStatistics, Institut Bergonié, Regional Cancer Center, 180 rue de Saint-Genès, Bordeaux Cedex, 33076, France; Unité INSERM 328, Bordeaux Cedex 33076, France

**Keywords:** non-Hodgkin’s lymphoma, residual disease, t(14;18), bcl-2, chemotherapy

## Abstract

Bcl-2, the gene over-expressed in follicular lymphomas (FL), is able to block chemotherapy-induced apoptosis. Consequently, we wondered whether bcl-2/IgH expression variations during treatment of FL could predict the outcome of patients with t(14;18)-bearing FL. For this purpose, we used a reverse transcription polymerase chain reaction (RT-PCR) assay to analyse 180 serial peripheral blood samples (PBS) during 34 treatment phases in 25 patients with t(14;18)-bearing FL. In all patients but two, bcl-2/IgH gene expression was demonstrated in pre-treatment samples. During 16 out of the 34 treatment phases (47%), bcl-2/IgH expression became negative: all but one were responders to chemotherapy. This conversion was transient in six cases. In 18 treatment phases, bcl2/IgH expression remained detectable: eight were clinically considered as treatment failures, while eight others achieved PR and two achieved CR. We observed a significant correlation between treatment response and RNA PCR results (*P* = 0.002). Three-year overall survival of patients with stable bcl2/IgH-negative conversion was 100% compared to 54% for the remaining patients (*P* = 0.069); 3-year freedom from progression was respectively 87.5% and 13% (*P* = 0.005). These results indicate a correlation between bcl-2/IgH expression variations and both clinical response and outcome. Whether this might predict disease outcome early remains to be confirmed. © 1999 Cancer Research Campaign


British Journal of Cancer (1999) **81**(5), 860–869
© 1999 Cancer Research Campaign
Article no. bjoc.1999.0777

### Evolution of BCL-2/IgH hybrid gene RNA expression during treatment of T(14;18)-bearing follicular lymphomas

**P Soubeyran[1], I Hostein[1], M Debled[2], H Eghbali[2], I Soubeyran[3], F Bonichon[4], T Astier-Gin[5] and B Hœrni[2]**

Departments of [1]Molecular Oncology, [2]Haematology, [3]Pathology and [4]Statistics, Institut Bergonié, Regional Cancer Center, 180 rue de Saint-Genès, 33076 Bordeaux Cedex, France; [5]Unité INSERM 328, 33076 Bordeaux Cedex, France

**Summary** Bcl-2, the gene over-expressed in follicular lymphomas (FL), is able to block chemotherapy-induced apoptosis. Consequently, we wondered whether bcl-2/IgH expression variations during treatment of FL could predict the outcome of patients with t(14;18)-bearing FL. For this purpose, we used a reverse transcription polymerase chain reaction (RT-PCR) assay to analyse 180 serial peripheral blood samples (PBS) during 34 treatment phases in 25 patients with t(14;18)-bearing FL. In all patients but two, bcl-2/IgH gene expression was demonstrated in pre-treatment samples. During 16 out of the 34 treatment phases (47%), bcl-2/IgH expression became negative: all but one were responders to chemotherapy. This conversion was transient in six cases. In 18 treatment phases, bcl2/IgH expression remained detectable: eight were clinically considered as treatment failures, while eight others achieved PR and two achieved CR. We observed a significant correlation between treatment response and RNA PCR results ($P = 0.002$). Three-year overall survival of patients with stable bcl2/IgH-negative conversion was 100% compared to 54% for the remaining patients ($P = 0.069$); 3-year freedom from progression was respectively 87.5% and 13% ($P = 0.005$). These results indicate a correlation between bcl-2/IgH expression variations and both clinical response and outcome. Whether this might predict disease outcome early remains to be confirmed. © 1999 Cancer Research Campaign

**Keywords:** non-Hodgkin's lymphoma; residual disease; t(14;18); bcl-2; chemotherapy

Follicular lymphoma (FL) is a well-defined entity among non-Hodgkin's lymphoma (NHL) and is associated with the t(14;18) that rearranges the bcl-2 gene with the immunoglobulin heavy-chain gene (IgH) (Cleary et al, 1986; Weiss et al, 1987). The hybrid gene, called bcl-2/IgH, produces hybrid mRNA and finally high constitutive levels of the normal bcl-2 protein (Tsujimoto and Croce, 1986). Consequently, the normal function of the bcl-2 gene should account for the behaviour of these tumours.

Indeed, bcl-2 blocks programmed cell death and immortalizes t(14;18)-bearing cells (Hockenbery et al, 1990), thus representing the first step before malignant transformation. It is probably responsible for the high rate of cells that remain in the G0 phase of the cell cycle (Linette et al, 1996; Mazel et al, 1996; O'Reilly et al, 1996; Vairo et al, 1996), hence explaining the slow growth pattern. Above all, recent studies have renewed the interest for bcl-2 while demonstrating its involvement in drug resistance (Alnemri et al, 1992; Kamesaki et al, 1993; Lotem and Sachs, 1993; Miyashita and Reed, 1993), which possibly accounts for the well-documented inability of conventional chemotherapy to cure disseminated disease. These results and in vitro studies that have demonstrated the possibility of reversal of bcl-2-induced resistance (Kitada et al, 1994; Reed et al, 1994; Keith et al, 1995) led us to study this problem in the specific context of FL.

We recently demonstrated the absence of bcl-2/IgH expression in t(14;18)-bearing cells circulating in patients with FL in complete remission (CR) (Soubeyran et al, 1993), suggesting that treatment may lead to bcl-2/IgH down-regulation. Consequently, we decided to explore this possible relationship between treatment and bcl-2/IgH down-regulation and to further investigate whether it could be related to treatment response and outcome of t(14;18)-bearing FL. We used our reverse transcription polymerase chain reaction (RT-PCR) technique to detect bcl-2/IgH RNAs (Soubeyran et al, 1993) in peripheral blood of FL patients.

*Received 7 October 1998*
*Revised 12 April 1999*
*Accepted 22 April 1999*

*Correspondence to:* P Soubeyran

### MATERIALS AND METHODS

### Patients

Patients selected in this study presented with a t(14;18)-bearing lymphoma as demonstrated by PCR performed on a representative lymph node biopsy. Pretreatment peripheral blood and bone marrow were not accepted as a valid source to account for the risk of tumour-unrelated benign t(14;18) clones (Limpens et al, 1991; Aster et al, 1992; Ohshima et al, 1993; Corbally et al, 1994; Liu et al, 1994; Bell et al, 1995; Ji et al, 1995; Limpens et al, 1995; Dolken et al, 1996; Fuscoe et al, 1996). Furthermore, although informed consent was requested, no sample was collected only for the study purpose. Consequently, there was no sample collection if no other blood analysis was needed. Finally, results were not made available to the physician during the patient's treatment.

During the study period (March 1992 to March 1997), 118 patients with FL tumours were treated either initially or at relapse at the Institut Bergonié. Frozen tumours were available in 62 of them and 36 were t(14;18)-bearing tumours (MBR breakpoints) as demonstrated by PCR analysis (Soubeyran et al, 1993). In all, 25 patients were considered for this study and are described in Table

**Table 1** Patient characteristics

| | | 25 patients | |
|---|---|---|---|
| Age | Median (range) | 46 (31–78) | |
| Centroblastic-centrocytic | Follicular | 17 | 68% |
| (Kiel classification) | Follicular and diffuse | 3 | 12% |
| | Diffuse | 1 | 4% |
| | With histologic transformation | 4 | 16% |
| Follicle centre lymphoma, follicular | Grade I | 9 | 36% |
| (REAL classification) | Grade II | 8 | 32% |
| | Grade III | 3 | 12% |
| | Diffuse | 1 | 4% |
| | With histologic transformation | 4 | 16% |
| Stage | I–II | 7 | 28% |
| | III–IV | 18 | 72% |
| Performance status (WHO scale) | 0–1 | 20 | 80% |
| | 2–4 | 5 | 20% |
| Bone marrow involvement | Yes | 11 | 44% |
| | No | 10 | 40% |
| | Missing | 4 | 16% |
| LDH | Abnormal | 5 | 20% |
| | Normal | 17 | 68% |
| | Missing | 3 | 12% |
| Treatment phase | Initial | 12 | 48% |
| | Relapse | 13 | 52% |

1. Histological diagnosis was confirmed as centroblastic-centrocytic lymphoma according to the Kiel classification (Lennert and Feller, 1992), and as follicle centre lymphoma according to the Revised European American Lymphoma (REAL) classification (Harris et al, 1994). Localized stages were treated by involved field irradiation either alone or combined with induction chemotherapy (CVP regimen) (Soubeyran et al, 1988), or low-dose total body irradiation as part of a prospective phase II trial (Richaud et al, 1998). After an eventual wait-and-see period, advanced stages were treated with different chemotherapies (Chauvergne et al, 1977; Bonadonna et al, 1985; Soubeyran et al, 1991) including continuous oral treatment with cyclophosphamide (100 mg per day) or chlorambucil (4 mg per day), CVP (cyclophosphamide 750 mg $m^{-2}$ day 1, vincristine 1.4 mg $m^{-2}$ day 1, prednisone 40 mg $m^{-2}$ days 1–7), fludarabine (25 mg $m^{-2}$ days 1–5), CEP (carmustine 100 mg $m^{-2}$ day 1, etoposide 100 mg $m^{-2}$ days 1–5, prednimustine 80 mg $m^{-2}$ days 1–5) or teniposide (100 mg $m^{-2}$ weekly). One patient received anti-CD52 monoclonal antibody therapy as part of a phase II study. Six patients had samples collected and analysed during successive treatment phases, allowing for analysis of 34 treatment phases in 25 patients.

### Methods

High molecular weight DNA and total cellular RNA were simultaneously extracted from frozen tumours with guanidine thiocyanate, caesium chloride and ultracentrifugation after grinding of the tumour in liquid nitrogen as previously described (Sambrook et al, 1989).

Peripheral mononuclear cells from peripheral blood samples (PBS) were separated on ficoll hypaque gradient. Then, nucleic acids were simultaneously extracted with the same method.

The PCR detection method of bcl-2/IgH DNA and transcripts have already been described (Soubeyran et al, 1993). We restricted the analysis to t(14;18) with breakpoints in the major breakpoint clustering region (MBR) using mbr3 and JH19 primers (Soubeyran et al, 1993) (Table 2).

**Table 2** Oligonucleotide primers and probe sequences

| | |
|---|---|
| Mbr3 | : 5′ TTT GAC CTT TAG AGA GTT GCT TTA CG 3′ |
| JH19 | : 5′ ACC TGA GGA GAC GGT GAC C 3′ |
| 18q21 probe | : 5′ CAC AGA CCC ACC CAG AGC CC 3′ |
| Raf 8 | : 5′ GAT GCA ATT CGA AGT CAC AGC G 3′ |
| Raf 9 | : 5′ TTT TCT CCT GGG TCC CAG ATA 3′ |
| Raf 9 probe | : 5′ GTC CAG TAG CCC CAA CAA TCT G 3′ |

PCR amplification of 1 μg DNA of each sample was performed in a total reaction volume of 50 μl (10 mM Tris–HCl pH 8.3, 50 mM potassium chloride (KCl), 2 mM magnesium chloride ($MgCl_2$), 200 μM dNTP each, primers 30 pmol each) as follows: hot-start PCR with a 94°C 5 min first step, then addition of one (tumour samples) or two units (PBS) of *Taq* polymerase (Perkin-Elmer Applied Biosystems Division, Foster City, CA, USA) followed by 35 (tumour samples) or 45 cycles (PBS) of the usual three phases (94°C denaturation step for 30 s, 55°C annealing step for 15 s and 72°C extension step for 45 s) in a thermal cycler (Perkin-Elmer Applied Biosystems Division).

Fifteen per cent of the PCR products were size-fractionated in a 2% NuSieve gel (FMC Bioproducts, Rockland, ME, USA), transferred to a Hybond N+ nylon membrane (Amersham, Buckinghamshire, UK), then hybridized with a 3′-end radiolabelled oligonucleotide probe 18q21 (Soubeyran et al, 1993) (Table 2) at 42°C overnight. Washing was carried out successively in 2 sodium–saline citrate (SSC)–0.1% sodium dodecyl sulphate (SDS) at room temperature, then 0.1 SSC–0.1% SDS at 50°C. Autoradiography was performed against a single intensifying screen at room temperature from one to 24 h.

The PCR sensitivity range was regularly monitored by dilution assays (Soubeyran et al, 1993). Furthermore, a highly diluted $10^{-4}$ positive control was inserted into each PCR assay to ensure that PCR could detect down to this level (Soubeyran et al, 1993). Quality of DNA samples was confirmed by c-raf-1 amplification at the DNA level.

*© 1999 Cancer Research Campaign* 

**Table 3** Sequence of the breakpoints of each clone

| | MBR breakpoint position[a] | N segment | JH segment breakpoint position[b] | PCR product size (bp) |
|---|---|---|---|---|
| 1 | 3057 | CCGACCCCTACCCT | 2363 JH5 | 141 |
| 2 | 3115 | GCGGCGGGGGCCCCAAGGTCGTCCAGCCGTCTCTGACATAGGACACACCGGCATGAACTAG | 2951 JH6 | 205 |
| 3 | 3129 | TGTCCTCGGGTCCGCA | 2949 JH6 | 234 |
| 4 | 3058 | GTCATGGGTATCCGTAGGGGCTGCTATCGGGCCCCATGGCTGGT | 2358 JH5 | 172 |
| 5 | 3065 | GGCCGGGACTCTGT | 2363 JH5 | 149 |
| 6 | 3054 | GATATTCC | 2956 JH6 | 144 |
| 7 | 3117 | ACCCCTCTTTGCCATCCT | 2955 JH6 | 218 |
| 8 | 3109 | GCCAACCTCGGGTGAT | 2952 JH6 | 211 |
| 9 | 3112 | TGCGCCT | 2952 JH6 | 205 |
| 10 | 3053 | CCCCATGACCAACAAC | 2950 JH6 | 157 |
| 11 | 3168 | GACGCGGGATA | 2953 JH6 | 264 |
| 12 | 3110 | CCGCGAGCAGAGTGTGGT | 2359 JH5 | 198 |
| 13 | 3165 | CGCAAGGATCCGGGCTTGATG | 2961 JH6 | 263 |
| 14 | 3115 | ATCACCCTC | 2963 JH6 | 199 |
| 15 | 3042 | CTTATC | 2960 JH6 | 126 |
| 16 | 3107 | AAAGGA | 1928 JH4 | 172 |
| 21 | 3052 | GCTACGTGTGTGATGTGGGCAAAGAAAA | 2955 JH6 | 163 |
| 23 | 3040 | CCTTAC | 2959 JH6 | 126 |
| 26 | 3110 | ACGGGCAAGACCTAAGTTTCCTCAACTTGA | 2951 JH6 | 228 |
| 27 | 3061 | AACGCCGACCCGGAGACCCAAAATCGCCTGGAGGCAACTGGT | 2357 JH5 | 171 |
| 31 | 3160 | no N segment | 2955 JH6 | 248 |
| 34 | 3164 | GAGGCCGGA | 2357 JH5 | 249 |
| 35 | 3056 | TCCCACTTA | 2950 JH6 | 153 |
| 36 | 3062 | CCGGCTGAGACCCCTTAGACTACTTTGACTA | 1924 JH4 | 154 |
| 38 | 3061 | CTGATAAGA | 1919 JH4 | 135 |

[a]According to Cleary published sequence (Cleary et al, 1986). [b]According to Ravetch published sequence (Ravetch et al, 1981).

To confirm further its specificity and uniqueness, we sequenced the PCR products obtained from the tumour DNA of the 15 patients. The sequencing reactions were performed directly from PCR-amplified DNA obtained with the same primers but 3′ tailed with –21M13 and reverse M13 sequences respectively on mbr3 and JH19. The PCR product was first purified using columns (Wizard PCR Preps resin DNA purification system®; Promega, Madison, WI, USA) to eliminate residual primers. Then, the sequence was performed according to the supplier's method with the ABI PRISM® Dye primer cycle sequencing kit and run on the automated DNA sequencer ABI prism 377 (Perkin-Elmer Applied Biosystems Division).

RNA samples were processed successively with DNAase I treatment, reverse transcription and standard PCR. Five micrograms of RNA were first treated by DNAase I (RNAase-free) in a total reaction volume of 25 µl: 50 mM Tris–HCl pH 8.3; 75 mM KCl; 3 mM $MgCl_2$; 1 mM dithiothreitol (DTT); 40 U RNasin (Promega); 70 U DNAase I RNAase-free (Boehringer, Mannheim, Germany). The reaction was incubated at 37°C for 1 h, then heated at 95°C for 5 min and immediately cooled on ice.

Then, 3 µg of these DNAase-treated RNA were submitted to reverse transcription. The reaction was performed in a total reaction volume of 50 µl in the following conditions: 50 mM Tris–HCl pH 8.3; 75 mM KCl; 3 mM $MgCl_2$; 1 mM dNTP each, 400 nM pd(N6) random hexamers (Life Technologies, Bethesda, MD, USA), 2 mM DTT, 80 U RNasin (Promega) and 200 U Superscript reverse transcriptase (Life Technologies). First, each sample without enzymes was denatured at 65°C for 5 min, then cooled on ice. Finally, enzymes and DTT were added and the reaction pursued at 37°C overnight.

For each sample, 3 different PCR reactions were simultaneously performed in three different tubes with the mbr3 and JH19 primers: 1 µg of DNA, cDNA generated from 1 µg of RNA using random hexamers, 1 µg of RNA without reverse transcription (as negative control to rule out DNA contamination still persisting after DNAase I treatment).

Further controls of these successive reactions were added: c-raf-1 gene amplification using raf8 and raf9 primers (as a control of both DNAase I and reverse transcription steps) as described previously (Table 2) (Soubeyran et al, 1993), and usual negative (negative DNA and no template) and diluted positive controls ($10^{-4}$ µg of positive DNA diluted in 1 µg of negative DNA). Each sample was analysed at least twice during different assays to ensure reliability of the results.

Finally, each sample was considered bcl-2/IgH RNA-positive (R+) only when the RNA control (without reverse transcription) was negative. Conversely, a sample was considered bcl-2/IgH RNA-negative (R–) only if the c-raf-1 RNA band was clearly detectable on agarose gel after ethidium bromide visualization.

We followed the recommendations of Kwok and co-workers (Kwok and Higuchi, 1989) concerning contamination control. Furthermore, the t(14;18) model with variable breakpoints and, consequently, variable PCR product lengths allowed for more reliability in contamination detection. If contamination occurred in any of the negative controls including RNA controls (without reverse transcription), the whole experiment was discarded.

 © 1999 Cancer Research Campaign

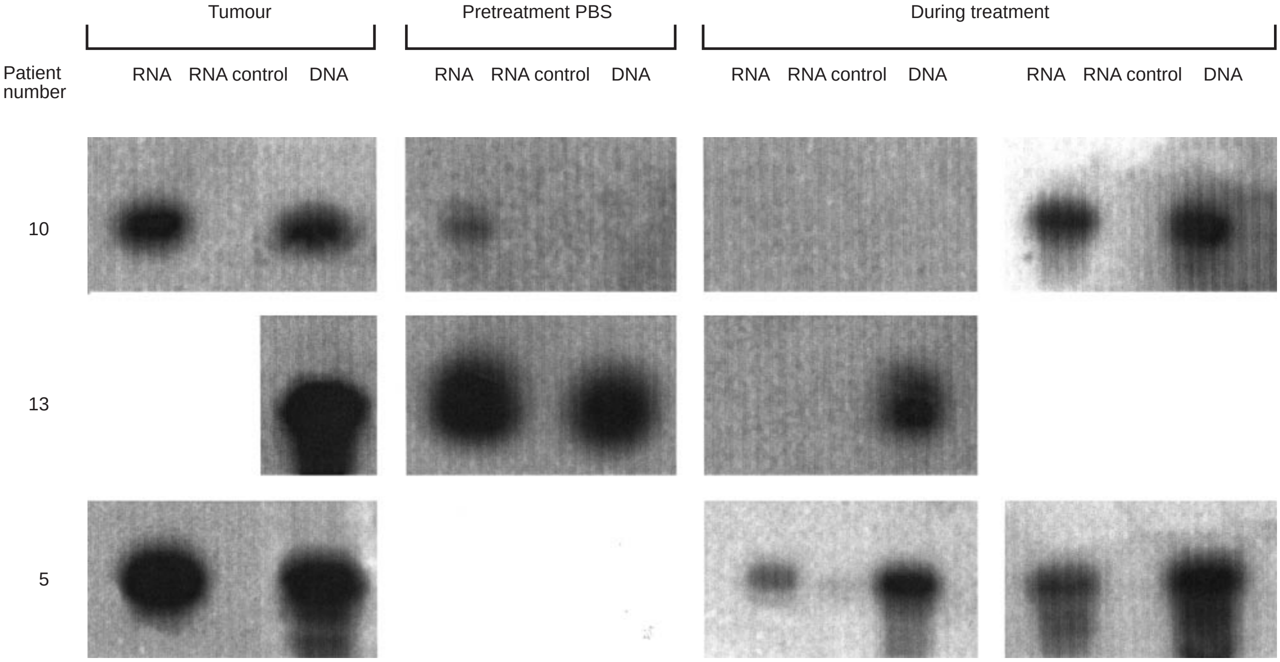

**Figure 1** PCR amplification of bcl-2/IgH hybrid gene at both DNA and RNA levels. Hybridization results with 18q21 probe of tumour and PBS either before or during treatment. Three lines per sample are represented: DNA for the PCR amplification of 1 μg of DNA; RNA for the PCR amplification of cDNA obtained after reverse transcription of 1 μg of RNA; RNA control for the PCR amplification of 1 μg of RNA without reverse transcription

### Statistical methods

The objective of the study was to predict response and outcome based on circulating disease patterns observed during treatment. Consequently, patterns of bcl-2/IgH RNA evolution were defined according only to results observed till the end of treatment.

Delay to PBS collection was computed from treatment initiation. Response was evaluated as usual with a minimum duration of 1 month to be acceptable (Miller et al, 1981). Relapse was considered as soon as the first symptom appeared. To study the relationship between qualitative variables, we used the $\chi^2$ test with Yates correction for $2 \times 2$ tables. Survival curves were computed with the Kaplan–Meier method and compared with the log-rank test. Overall survival was computed from treatment initiation to death or date last known alive. All causes of death were considered as events. Freedom from progression was computed from treatment initiation to progression or date last known free of disease, considering progression as an event. In patients with two or more successive treatment phases, survival curves were computed from initiation of the first one.

The cut-off date for the last evaluation was 23 March 1998. Median follow-up computed from treatment initiation to cut-off date is 46.5 months (range 9–126 months).

### RESULTS

### Tumour and pretreatment PBS analysis

The t(14;18) tumour clone was clearly identified in all 25 cases. The specificity and uniqueness of the PCR products was confirmed through internal probe hybridization and sequencing of the PCR products (Table 3).

Pretreatment bcl-2/IgH gene RNA expression in tumour cells was confirmed in all but two cases, either in tumour (nine patients), pretreatment blood sample (five patients) or both (nine patients).

A total of 235 PBS were collected. Fifty-five PBS results (23%) were not conclusive because of poor RNA quality, as demonstrated by the absence of c-raf-1 RNA amplification. Thus, results of 180 PBS were analysed, 51 collected before treatment initiation and 129 during and after treatment. The median number of PBS analysed per patient was seven (range 1–15). Results are outlined in Tables 4–6. Representative samples are presented in Figure 1.

### Analysis of PBS during treatment

The evolution of bcl2/IgH expression in t(14;18)-bearing cells was investigated during treatment. There was a negative conversion of bcl-2/IgH expression, as defined by complete disappearance of the specific RNA signal in the circulating cells, during 16 treatment phases (47%) in 14 patients. Three patterns of evolution were observed: positive on each sample (18 treatment phases) and negative conversion (16 treatment phases) that could be transient (six out of 16 phases) (Tables 4–6).

Bcl-2/IgH expression was always detectable in 18 treatment phases (15 patients) (Tables 4 and 7): no treatment response was observed in eight patients, while ten responded to chemotherapy, eight of them partially. Among responding patients, six later presented disease progression from 5 to 66 months after treatment initiation (median 16 months), two relapsed after CR achievement at 7 and 11 months and two patients remained in stable PR at 7.5 and 59 months.

In ten treatment phases (nine patients), negative conversion was observed until the end of treatment (patient numbers 10–2, 11, 12,

**Table 4** Bcl-2/IgH RNA expression during treatment. Patients who remained PBS bcl2/IgH RNA positive during treatment

| Patient no. | Stage | Treatment | Reference[a] | Response | Delay[b] (months) | Pbs Status | Evolution[c] |
|---|---|---|---|---|---|---|---|
| 1 | III | CEP | D+R+(Tm) | failure | 1 | D+R+ | Death 7 m. later |
| 2 | II | Irradiation | D+R+(Tm) | no change | 0.5 | D+R+ | |
| | | | | failure | 1 | D+R+ | Death 1 m. later |
| 3 | IV | MoAb | D+(Tm) | failure | 1.5 | D+R+ | Death 2.5 m. later |
| 4 | III | CVP | D+R+(Tm) | no change | 6 | D+R+ | |
| | | | | failure | 6.5 | | Death 0.5 m. later |
| 5 | IV | CPA | D+R+(Tm) | PR < 50% | 5.5 | D+R+ | |
| 1st treatment phase | | CPA | | PR = 50% | 22 | D+R+ | |
| | | CPA | | PR = 50% | 23 | D+R+ | |
| | | CPA | | PR = 50% | 23.5 | D+R+ | |
| | | CPA | | failure | 26 | D+R+ | Prog. 0 m. |
| 5 | IV | Tenip. | | failure | 2 | D+R+ | Death 0.5 m. later |
| 2nd treatment phase | | | | | | | |
| 6 | III | CVP | D+R+(Tm+pPBS) | PR > 50% | 1 | D+R+ | |
| | | CVP | | PR > 50% | 2 | D+R+ | |
| | | CVP | | PR > 50% | 3 | D+R+ | |
| | | CVP | | PR > 50% | 4 | D–R+ | |
| | | | | failure | 7 | | Death 2 m. later |
| 7 | II | CVP | D+R+(Tm+pPBS) | PR = 50% | 2 | D+R+ | |
| 1st treatment phase | | CVP | | CR | 3 | | Relapse 4 m. later |
| 9 | III | CHB | D+(Tm) | PR > 75% | 55 | D–R+ | |
| | | | | failure | 66 | | Death 2 m. later |
| 14 | IV | CVP | D+R+(Tm+pPBS) | PR > 50% | 2 | D+R+ | |
| 1st treatment phase | | CVP | | PR > 50% | 3 | D+R+ | |
| | | CVP | | PR > 50% | 8 | D+R+ | Prog. 3 m. |
| 15 | IV | CVP | D+R+(Tm+pPBS) | no change | 1 | D+R+ | |
| 1st treatment phase | | CVP | | no change | 2 | D+R+ | Prog. 2 m. |
| 15 | III | CEOP | | failure | 4 | D+R+ | Death 3 m. |
| 3rd treatment phase | | | | | | | |
| 26 | III | Fluda. | | MR | 1 | D+R+ | |
| 2nd treatment phase | Fluda. | | MR | 3 | D+R+ | | |
| | | Fluda. | | MR | 6 | R+ | |
| | | | | progression | 11 | | Death 9 m. later |
| 34 | IV | CVP | D+R+(Tm) | PR | 1 | D+R+ | |
| | | CVP | | PR | 3 | D+R+ | |
| | | | | PR | 3.5 | D+R+ | |
| | | | | progression | 5 | | Alive in prog. 11 m. |
| 35 | III | CVP | D+(Tm)D+R+(pPBS) | PR | 1 | D+R+ | |
| | | CVP | | PR | 2 | D–R+ | NED 5.5 m. |
| 36 | III | CEOP | D+(Tm)D+R+(pPBS) | PR | 0.7 | D+R+ | |
| 1st treatment phase | | CEOP | | PR | 2 | D+R+ | |
| | | CEOP | | PR | 3 | D+R+ | |
| | | CEOP | | CR | 4 | D+R+ | |
| | | No treatment | | CR | 8 | D+R+ | Relapse 3 m. later |
| 36 | III | CPA | | PR | 6 | D+R+ | |
| 2nd treatment phase | | CPA | | PR | 12 | D+R+ | |
| | | CPA | | PR | 16 | D+R+ | |
| | | | | progression | 16.5 | | Death 4 m. later |
| 38 | IV | CVP | D+R+(Tm+pPBS) | PR | 1 | D+R+ | |
| | | | | uCR | 41 | D+R+ | NED 18 m. later |

[a]Reference sample for bcl-2/IgH RNA expression. Tm: tumour – pPBS: pretreatment peripheral blood sample – D: t(14;18) PCR result at the DNA level – R: t(14;18) PCR result at the RNA level. [b]Delay from treatment initiation to PBS collection. [c]Evolution since the last PBS collection. CVP: cyclophosphamide, vincristine, prednisone; CEP: lomustine, etoposide, prednimustine; CPA: cyclophosphamide; CHB: chlorambucil; Tenip.: teniposide; Fluda.: fludarabine; MoAb: monoclonal antibody; PR: partial response; CR: complete response; prog.: progression; AsPR: alive in stable PR; NED: no evidence of disease.

13, 14–2, 23 – Table 5; 10–1, 16, 21, 31 – Table 6); nine responses were observed, including eight CR. The patient in PR relapsed at 35 months after treatment initiation, while the eight CR remained disease-free 11–87 months after treatment initiation (median 38 months). The remaining treatment phase was associated with progression: the phenotype D–R– was observed twice.

In six treatment phases (five patients), bcl-2/IgH became undetectable transiently (one isolated R– PBS) then returned to positive (patient numbers 7–2, 7–3, 8, 26, 27 – Table 5; 15 – Table 6): all these responded to treatment. Four patients relapsed at 16–35 months after treatment initiation and the last remains in stable PR at 24 months.

### Correlation between bcl-2/IgH expression and treatment response

In one patient with two successive treatments (No. 14), bcl-2/IgH expression remained positive at a constant level during CVP chemotherapy (Table 4). Progression occurred 3 months after CVP discontinuation and fludarabine was given at this time. During this

 *© 1999 Cancer Research Campaign*

**Table 5** Bcl-2/IgH RNA expression during treatment. Confirmed negative conversion during treatment: D+R– phenotype

| Patient no. | Stage | Treatment | Reference[a] | Response | Delay[b] (months) | Pbs Status | Evolution[c] |
|---|---|---|---|---|---|---|---|
| 7 | IV | CVP | D+R+(pPBS) | PR > 50% | 1 | D+R– | |
| 2nd treatment phase | | CVP | | PR > 75% | 4 | D+R+ | |
| 7 | | CPA | | PR > 75% | 10 | D+R– | |
| 3rd treatment phase | | CPA | | PR > 75% | 24 | D+R+ | AsPR |
| 8 | IV | CVP | D+(Tm) D+R+(pPBS) | PR > 50% | 1 | D–R+ | |
| | | CVP | | PR > 50% | 2 | D–R+ | |
| | | CVP | | PR > 50% | 3 | D+R– | |
| | | CVP | | PR > 50% | 4 | D–R+ | |
| | | CVP | | PR > 50% | 6 | D+R+ | |
| | | No treatment | | PR > 50% | 7 | D+R+ | |
| | | | | PR > 50% | 9 | D+R– | |
| | | | | PR > 50% | 11 | D+R+ | |
| | | | | PR > 50% | 18 | D+R+ | |
| | | | | PR > 50% | 27 | D+R+ | Relapse 3 m. AsPR 2 m. later |
| 10 | | CPA | | PR | 2 | D–R– | |
| 2nd treatment phase | | CPA | | PR | 10 | D+R– | |
| | | CPA | | PR | 10.5 | D–R+ | |
| | | CPA | | PR | 12.5 | D–R– | |
| | | CPA | | PR | 19 | D–R– | |
| | | No treatment | | PR | 28 | D+R+ | Relapse 7 m. Death 9 m. later |
| 11 | IV | Tenip. | D+(Tm) | PR > 50% | 1 | D+R+ | |
| | | Tenip. | D+R+(pPBS) | PR > 50% | 14 | D–R+ | |
| | | Tenip. | | PR > 50% | 17 | D+R+ | |
| | | Tenip. | | CR | 24 | D+R– | |
| | | Tenip. | | CR | 25 | D+R– | |
| | | Tenip. | | CR | 28 | D–R– | |
| | | Tenip. | | CR | 31 | D+R– | |
| | | No treatment | | CR | 34 | D+R+ | |
| | | | | CR | 55 | D–R– | |
| | | | | CR | 59 | D–R– | NED 28 m. |
| 12 | IV | Fluda. | D+R+(Tm+pPBS) | PR > 75% | 3 | D+R+ | |
| | | Fluda. | | PR > 75% | 4 | D–R+ | |
| | | Fluda. | | CR | 8 | D–R– | |
| | | No treatment | | CR | 10 | D+R– | |
| | | | | CR | 14 | D+R– | |
| | | | | CR | 21 | D–R+ | |
| | | | | CR | 31 | D+R– | NED 4 m. |
| 13 | III | CVP | D+R+(Tm+pPBS) | CR | 0.7 | D+R– | |
| | | CVP | | CR | 2 | D–R– | |
| | | No treatment | | CR | 4 | D+R– | |
| | | | | CR | 7 | D+R– | |
| | | | | CR | 21 | D–R– | NED |
| 14 | IV | Fluda. | D+R+(Tm+pPBS) | PR | 1 | D+R+ | |
| 2nd treatment phase | | Fluda. | | CR | 3 | R+ | |
| | | Fluda. | | CR | 4 | D+R– | |
| | | | | CR | 22 | D+R+ | NED 4 m. |
| 26 | II | CVP | D+R+(Tm) | PR > 50% | 2 | D+R– | |
| 1st treatment phase | | CVP | | CR | 3 | D+R+ | Relapse III 13 m. Death 25 m. later |
| 27 | II | L85 | D+(Tm) D+R+(pPBS) | NE | 2 days | D+R+ | |
| | | L85 | | NE | 4 days | D+R– | |
| | | L85 | | PR > 50% | 1 | D+R+ | |
| | | L85 | | PR > 50% | 1.1 | D+R+ | |
| | | | | CR | 4 | D–R– | |
| | | | | CR | 8 | D–R– | |
| | | | | CR | 11 | D+R+ | |
| | | | | | 18 | D+R+ | Relapse II 0 m. NED 6 m. |

See Table 4 legend for abbreviations and key; L85 = phase II trial in localized FL with induction low-dose total body irradiation followed by involved field irradiation.

second treatment phase, circulating t(14;18)-bearing cells rapidly became bcl-2/IgH RNA negative and the patient achieved CR (Table 5). He remained in persistent CR 26 months after second treatment initiation.

We observed a significant relationship between treatment response and RNA PCR results (Table 7) ($P$ = 0.002). Among 13 complete responses, negative conversion of bcl-2/IgH expression was observed in 11 cases during treatment. The only patient with

**Table 6** Bcl-2/IgH RNA expression during treatment. Unconfirmed negative conversion during treatment: D–R– phenotype

| Patient no. | Stage | Treatment | Reference[a] | Response | Delay[b] | Pbs Status | Evolution[c] |
|---|---|---|---|---|---|---|---|
| 10 | III | CVP | D+R+(Tm) | PR | 0.7 m. | D–R– | |
| 1st treatment phase | | CVP | D–R+(pPBS) | failure | 1.5 m. | D–R– | Death 44 m. later |
| 15 | IV | CPA | | PR < 50% | 3 m. | D+R+ | |
| 2nd treatment phase | | CPA | | PR > 50% | 11 m. | D+R+ | |
| | | CPA | | PR > 50% | 15 m. | D–R– | |
| | | CPA | | CR | 21 m. | D–R+ | |
| | | CPA | | CR | 25 m. | D+R+ | |
| | | | | CR | 28 m. | D–R– | Relapse III 7 m. |
| 16 | II | L85 | D+R+(Tm+pPBS) | CR | 3 m. | D+R+ | |
| | | L85 | | CR | 5 m. | R– | |
| | | | | CR | 9 m. | D+R+ | |
| | | | | CR | 17.5 m. | R+ | |
| | | | | CR | 41 m. | D–R– | NED 6 m. |
| 21 | I | L85 | D+R+(Tm) | PR | 2 d. | D+R+ | |
| | | L85 | | PR | 4 d. | D+R+ | |
| | | L85 | | PR | 1 m. | D–R– | |
| | | L85 | | PR | 1.1 m. | D–R– | |
| | | | | CR | 3 m. | D–R+ | NED 8 m. |
| 23 | IV | Fluda. | D+R+(Tm) | PR | 1 m. | D–R– | |
| | | Fluda. | | PR | 3 m. | D–R– | |
| | | Fluda. | | CR | 5 m. | D–R– | |
| | | | | CR | 13 m. | D–R+ | |
| | | | | CR | 16 m. | D–R– | |
| | | | | CR | 18 m. | D–R– | |
| | | | | CR | 20 m. | D–R– | |
| | | | | CR | 23 m. | D+R– | |
| | | | | CR | 26 m. | D–R+ | |
| | | | | CR | 32 m. | D–R– | NED 13 m. |
| 31 | I | Irradiation | D+R+(Tm) | CR | 3 m. | D–R– | |
| | | | | CR | 5 m. | D–R– | |
| | | | | CR | 11 m. | D+R+ | |
| | | | | CR | 31 m. | D+R+ | NED 11 m. |

See Table 5 legend for abbreviations and key.

association of negative conversion and treatment failure (no. 10, Table 6) had the D–R– phenotype.

### Correlation between bcl-2/IgH expression and survival

To analyse overall survival and freedom from progression in these patients, we considered only the first treatment phase of each treatment. We designated two groups, i.e. patients with t(14;18)-bearing tumour cells who either remained RNA-positive during treatment or became transiently negative (R+ group) and those whose tumour cells became RNA-negative till the end of treatment (R– group). Median follow-up of these two groups is respectively 46.5 and 47 months (range 9–126 and 9–68 months). Three-year overall survival of the R+ and R– groups was respectively 54% and 100% (Figure 2, $P$ = 0.069). Three-year freedom from progression of the R+ and R– groups was respectively 13% and 87.5% (Figure 2, $P$ = 0.005).

### PBS analysis at the DNA level

The same patterns of evolution were observed at the DNA level: always positive (21 treatment phases, 14 patients), and negative conversion (13 treatment phases, 13 patients) that could be transient (three treatment phases, three patients). No significant relationship between DNA results and treatment response was observed (Table 7, $P$ = 0.27). We then analysed overall survival and freedom from progression in these patients, considering only PCR results obtained during the first treatment phase of each patient. We designated two groups, i.e. patients with stable disappearance of t(14;18)-bearing tumour cells during treatment (D–) and those with either persistence or transient disappearance of tumour cells (D+). No significant difference was observed between the two groups for overall survival (Figure 3, $P$ = 0.317). Freedom from progression was significantly higher in the D– group (Figure 3, $P$ = 0.032).

### DISCUSSION

Recently, it was demonstrated that, as expected, benign t(14;18) clones can circulate in the blood of healthy patients (Bell et al, 1995; Ji et al, 1995; Limpens et al, 1995; Dolken et al, 1996; Fuscoe et al, 1996). Moreover, multiple independent bcl-2/JH translocations may simultaneously be present in the blood of healthy donors (Ji et al, 1995; Limpens et al, 1995; Fuscoe et al, 1996). Although non-malignant, these circulating clones have been suspected to be the first step that might later lead to FL occurrence in some, probably rare, cases (Liu et al, 1994; Ji et al, 1995). Indeed, multiple circulating t(14;18)-bearing clones have been observed in the blood of some FL patients (Price et al, 1991), suggesting that malignant and benign t(14;18) clones co-exist. Consequently, the t(14;18)-bearing clones detected in the blood of FL patients could be either malignant or benign.

To pursue the study of residual disease in FL, methods must be found to ensure that the detected clones are truly malignant. Two

 *© 1999 Cancer Research Campaign*

**Table 7** Relationship between treatment response and evolution of circulating disease patterns during treatment: bcl-2/IgH expression and standard DNA PCR

| | No complete response | Complete response | *P*-value[a] |
|---|---|---|---|
| Remaining R+ during treatment | 16 | 2 | |
| Negative conversion R+ to R– | 5 | 11 | 0.002 |
| Remaining D+ during treatment | 15 | 6 | |
| Negative conversion D+ to D– | 6 | 7 | 0.27 |

[a]Chi-square with Yates correction.

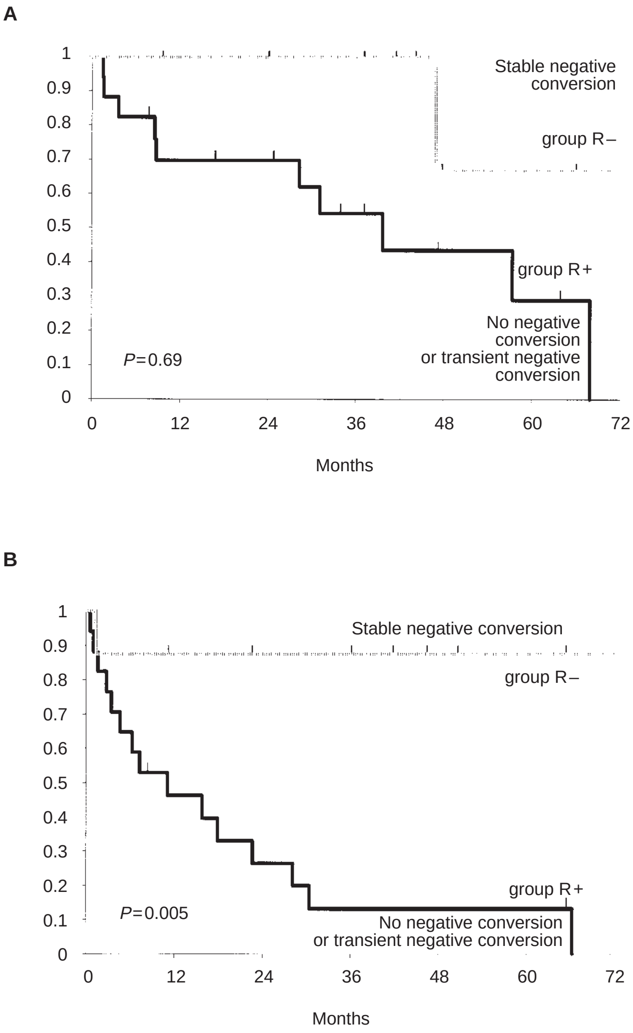

**Figure 2** Patients with either persistence or transient disappearance of bcl-2/IgH expression in circulating tumour cells during treatment (group R+) and patients with stable disappearance of bcl-2/IgH expression in circulating tumour cells during treatment (group R–); (**A**) overall survival; (**B**) freedom from progression possible solutions might offset this problem. The first derives from quantitative analyses performed in healthy patients (Liu et al, 1994; Ji et al, 1995; Dolken et al, 1996; Fuscoe et al, 1996). Such studies have demonstrated that, in more than 90% of cases, the amount of benign circulating cells is below the $10^{-5}$ sensitivity limit of usual PCR assays. Furthermore, Dolken showed that the amount of circulating cells was significantly lower in healthy donors than in localized FL in long-term persistent CR (Dolken et al, 1996). Therefore, PCR sensitivity must be controlled and kept above the $10^{-5}$ threshold.

Another possibility is to identify the FL-related t(14;18) clone in tumour material, so as to trace it further in the blood of FL patients, while ignoring other unrelated clones. Although neither of these methods absolutely eliminates the risk of misinterpreting a benign clone as malignant, it is no longer possible to pursue residual studies in the FL model without the mandatory use of both of these methods. Therefore, we decided to select patients with t(14;18)-bearing tumours that were clearly tumour-related as demonstrated on a representative tumour sample, thus excluding PBS and bone marrow as a source. Furthermore, we sequenced each clone to ensure its uniqueness and precisely monitored the PCR sensitivity range.

In a previous study, we designed an original PCR-based method that allowed for the detection of bcl-2/IgH RNA in t(14;18)-bearing cells (Soubeyran et al, 1993). Preliminary results showed that bcl-2/IgH expression could not be detected in t(14;18)-bearing circulating cells in a significant proportion of patients in CR. However, no serial analysis was done to demonstrate the probable relationship between bcl-2/IgH RNA disappearance and treatment.

In the present series, we analysed 180 PBS from 25 patients during 34 treatment phases with previous pretreatment samples in most of the cases. In 16 treatment phases (47%), we demonstrated the disappearance of bcl-2/IgH RNAs, thus confirming the expected influence of treatment on bcl-2/IgH gene expression.

The correlation of bcl-2/IgH expression evolution with response to treatment was significant. Among the 13 patients who achieved CR, only two were not associated with expression down-regulation. Conversely, as expected, the absence of negative conversion was associated with partial responses or even failures in most of the cases (16 out of 18).

We further wondered whether these results could be related to outcome and noted significant improvement of progression-free survival in patients whose circulating cells remained bcl-2/IgH RNA negative till the end of treatment. A similar tendency was observed for overall survival, although it was not significant. The only patient with adverse outcome in the R– group (no. 10, Table 6) had a D–R– phenotype, which does not strictly demonstrate that circulating cells became bcl-2/IgH-negative: such cells could remain undetectable, below the PCR sensitivity threshold, and still be bcl-2/IgH RNA positive. Nonetheless, this patient was considered as a negative conversion.

This relationship between down-regulation of bcl-2/IgH expression and outcome was expected because of the correlation observed with response that is already known to have important prognostic value (Soubeyran et al, 1991). However, this is another

© 1999 Cancer Research Campaign

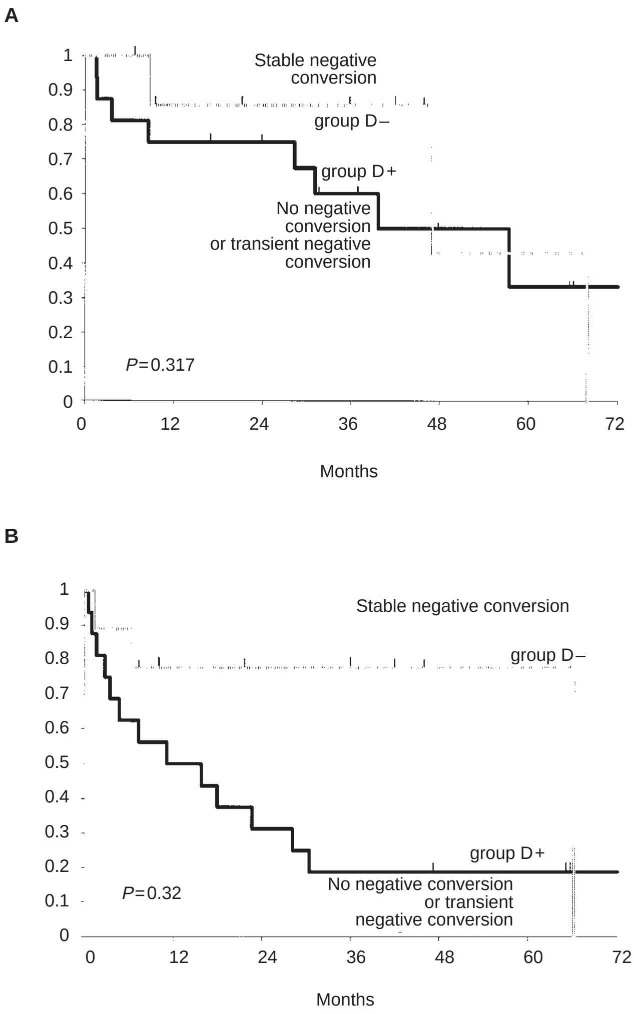

**Figure 3** Patients with either persistence or transient disappearance of circulating t(14;18)-bearing cells during treatment (group D+) and patients with stable disappearance of circulating t(14;18)-bearing cells during treatment (group D–); (**A**) overall survival; (**B**) freedom from progression argument for the potential value of bcl-2/IgH expression down-regulation in FL. This result points to new opportunities for FL treatment. Indeed, bcl-2 down-regulation might not only be a tool to assess early the potential value of a new treatment approach but also could represent an aspect of future treatment strategies.

Our results are further corroborated by recent knowledge on the ability of bcl-2 to block programmed cell death (Hockenbery et al, 1990) and consequently chemotherapy-induced apoptosis in cancer cells. Since Miyashita's early results (Miyashita and Reed, 1992, 1993), many authors have confirmed the importance of bcl-2 in drug resistance (Alnemri et al, 1992; Kamesaki et al, 1993; Lotem and Sachs, 1993) and later, the possibility to reverse this phenomenon by antisense oligonucleotides directed against bcl-2 mRNA (Kitada et al, 1994; Reed et al, 1994; Keith et al, 1995). Our clinical results are clearly in accordance with such studies.

We also analysed our results at the DNA level. No significant difference was observed between DNA results observed during treatment and response or overall survival. Yet a significant freedom from progression advantage was demonstrated in favour of patients whose circulating cells disappeared during treatment, as expected according to Gribben's studies (Gribben et al, 1991; Gribben, 1994). It should be pointed out that we considered results only obtained during treatment but not subsequent samples, since we were searching for a predictive test available at the end of treatment, a moment when useful therapeutic options could be discussed. However, the quantitative analysis of these results would be more appropriate and probably would improve the predictive value of DNA PCR in this setting.

In conclusion, we have demonstrated a relationship between treatment and bcl-2/IgH expression down-regulation. Furthermore, RNA-negative conversion was significantly correlated with CR and was associated with a lower rate of relapse or progression. These results could be considered as a confirmation of in vitro data showing that bcl-2 is involved in drug resistance. Finally, bcl-2/IgH expression analysis offers new opportunities to take advantage of the FL model while analysing the activity of the oncogene that is specifically disregulated in tumour cells. However, whether bcl-2/IgH expression monitoring will be of clinical value during treatment of FL patients remains to be demonstrated in further prospective studies. Finally, as new treatment strategies become available in FL (Solal-Celigny et al, 1993; Rohatiner et al, 1994; Freedman et al, 1996; McLaughlin et al, 1996; Bierman et al, 1997), this new tool could help to define whether these treatments really influence the biological behaviour of FL, thus explaining further potential clinical improvements. It could also help in the search for new agents that might potentially alter the currently unaffected FL natural history.

### ACKNOWLEDGEMENTS

We are most grateful to Jean-Luc Birac for the graphical preparation. Special thanks to Dorothée Quincy for the preparation of the manuscript. Supported by grants from the Ligue Nationale contre le Cancer, Comités Départementaux de la Gironde, des Pyrénées Atlantiques et des Landes de la Ligue Nationale contre le Cancer, Fédération des GEFLUC.

### REFERENCES

Alnemri ES, Fernandes TF, Haldar S, Croce CM and Litwack G (1992) Involvement of BCL-2 in glucocorticoid-induced apoptosis of human pre-B-leukemias. *Cancer Res* **52**: 491–495

Aster JC, Kobayashi Y, Shiota M, Mori S and Sklar J (1992) Detection of the t(14;18) at similar frequencies in hyperplastic lymphoid tissues from American and Japanese patients. *Am J Pathol* **141**: 291–299

Bell DA, Liu Y and Cortopassi GA (1995) Occurrence of bcl-2 oncogene translocation with increased frequency in the peripheral blood of heavy smokers. *J Natl Cancer Inst* **87**: 223–224

Bierman PJ, Vose JM, Anderson JR, Bishop MR, Kessinger A and Armitage JO (1997) High-dose therapy with autologous hematopoietic rescue for follicular low-grade non-Hodgkin's lymphoma. *J Clin Oncol* **15**: 445–450

Bonadonna G, Viviani S, Valagussa P, Bonfante V and Santoro A (1985) Third-line salvage chemotherapy in Hodgkin's disease. *Semin Oncol* **12** (suppl 2): 23–25

Chauvergne J, Durand M, Hoerni B, Hoerni-Simon G, Brunet R and Lagarde C (1977) Induction chemotherapy of non-Hodgkin's malignant lymphomas. Preliminary results of a controlled trial. *Eur J Cancer* **13**: 399–400

Cleary ML, Smith SD and Sklar J (1986) Cloning and structural analysis of cDNAs for bcl-2 and a hybrid bcl-2/immunoglobulin transcript resulting from the t(14;18) translocation. *Cell* **47**: 19–28

Corbally N, Grogan L, Keane MM, Devaney DM, Dervan PA and Carney DN (1994) Bcl-2 rearrangement in Hodgkin's disease and reactive lymph nodes. *Am J Clin Pathol* **101**: 756–760

Dolken G, Illerhaus G, Hirt C and Mertelsmann R (1996) BCL-2/J(H) rearrangements in circulating B cells of healthy blood donors and patients with non-malignant diseases. *J Clin Oncol* **14**: 1333–1344

© 1999 Cancer Research Campaign

Freedman AS, Gribben JG, Neuberg D, Mauch P, Soiffer RJ, Anderson KC, Pandite L, Robertson MJ, Kroon M, Ritz J and Nadler LM (1996) High-dose therapy and autologous bone marrow transplantation in patients with follicular lymphoma during first remission. *Blood* **88**: 2780–2786

Fuscoe JC, Setzer RW, Collard DD and Moore MM (1996) Quantification of t(14;18) in the lymphocytes of healthy adult humans as a possible biomarker for environmental exposures to carcinogens. *Carcinogenesis* **17**: 1013–1020

Gribben JG (1994) Attainment of molecular remission: a worthwhile goal? *J Clin Oncol* **12**: 1532–1534

Gribben JG, Freedman AS, Neuberg D, Roy DC, Blake KW, Woo SD, Grossbard ML, Rabinowe SN, Coral F, Freeman GJ, Ritz J and Nadler LM (1991) Immunologic purging of marrow assessed by PCR before autologous bone marrow transplantation for B-cell lymphoma. *N Engl J Med* **325**: 1525–1533

Harris NL, Jaffe ES, Stein H, Banks PM, Chan JK, Cleary ML, Delsol G, De Wolf Peeters C, Falini B, Gatter KC, Grogan TM, Isaacson PG, Knowles DM, Mason DY, Muller-Hermelink HK, Pileri SA, Piris MA, Ralfkiaer E and Warnke RA (1994) A revised European-American classification of lymphoid neoplasms: a proposal from the International Lymphoma Study Group. *Blood* **84**: 1361–1392

Hockenbery D, Nunez G, Milliman C, Schreiber RD and Korsmeyer SJ (1990) Bcl-2 is an inner mitochondrial membrane protein that blocks programmed cell death. *Nature* **348**: 334–336

Ji WZ, Qu GZ, Ye P, Zhang XY, Halabi S and Ehrlich M (1995) Frequent detection of bcl-2/J(H) translocations in human blood and organ samples by a quantitative polymerase chain reaction assay. *Cancer Res* **55**: 2876–2882

Kamesaki S, Kamesaki H, Jorgensen TJ, Tanizawa A, Pommier Y and Cossman J (1993) Bcl-2 protein inhibits etoposide-induced apoptosis through its effects on events subsequent to topoisomerase II-induced DNA strand breaks and their repair. *Cancer Res* **53**: 4251–4256

Keith FJ, Bradbury DA, Zhu YM and Russell NH (1995) Inhibition of bcl-2 with antisense oligonucleotides induces apoptosis and increases the sensitivity of AML blasts to Ara-C. *Leukemia* **9**: 131–138

Kitada S, Takayama S, De Riel K, Tanaka S and Reed JC (1994) Reversal of chemoresistance of lymphoma cells by antisense-mediated reduction of bcl-2 gene expression. *Antisense Res Dev* **4**: 71–79

Kwok S and Higuchi R (1989) Avoiding false positive with PCR. *Nature* **339**: 237–238

Lennert K and Feller AC (1992) Histopathology of non-Hodgkin's lymphomas. Springer-Verlag: Berlin

Limpens J, de Jong D, van Krieken JH, Price CG, Young BD, van Ommen GJ and Kluin PM (1991) Bcl-2/JH rearrangements in benign lymphoid tissues with follicular hyperplasia. *Oncogene* **6**: 2271–2276

Limpens J, Stad R, Vos C, de Vlaam C, de Jong D, van Ommen GJ, Schuuring E and Kluin PM (1995) Lymphoma-associated translocation t(14;18) in blood B cells of normal individuals. *Blood* **85**: 2528–2536

Linette GP, Li Y, Roth K and Korsmeyer SJ (1996) Cross talk between cell death and cell cycle progression: BCL-2 regulates NFAT-mediated activation – inaugural paper. *Proc Natl Acad Sci USA* **93**: 9545–9552

Liu Y, Hernandez AM, Shibata D and Cortopassi GA (1994) BCL2 translocation frequency rises with age in humans. *Proc Natl Acad Sci USA* **91**: 8910–8914

Lotem J, Sachs L (1993) Regulation by bcl-2, c-myc, and p53 of susceptibility to induction of apoptosis by heat shock and cancer chemotherapy compounds in differentiation-competent and -defective myeloid leukemic cells. *Cell Growth Differ* **4**: 41–47

McLaughlin P, Hagemeister FB, Romaguera JE, Sarris AH, Pate O, Younes A, Swan F, Keating M and Cabanillas F (1996) Fludarabine, mitoxantrone, and dexamethasone: An effective new regimen for indolent lymphoma. *J Clin Oncol* **14**: 1262–1268

Mazel S, Burtrum D and Petrie HT (1996) Regulation of cell division cycle progression by bcl-2 expression: a potential mechanism for inhibition of programmed cell death. *J Exp Med* **183**: 2219–2226

Miller AB, Hoogstraten B, Staquet M and Winkler A (1981) Reporting results of cancer treatment. *Cancer* **47**: 207–214

Miyashita T and Reed JC (1992) Bcl-2 gene transfer increases relative resistance of S49.1 and WEH17.2 lymphoid cells to cell death and DNA fragmentation induced by glucocorticoids and multiple chemotherapeutic drugs. *Cancer Res* **52**: 5407–5411

Miyashita T and Reed JC (1993) Bcl-2 oncoprotein blocks chemotherapy-induced apoptosis in a human leukemia cell line. *Blood* **81**: 151–157

O'Reilly LA, Huang DCS and Strasser A (1996) The cell death inhibitor Bcl-2 and its homologues influence control of cell cycle entry. *EMBO J* **15**: 6979–6990

Ohshima K, Kikuchi M, Kobari S, Masuda Y, Eguchi F and Kimura N (1993) Amplified bcl-2/JH rearrangements in reactive lymphadenopathy. *Virchows Arch B Cell Pathol Incl Mol Pathol* **63**: 197–198

Price CG, Tuszynski A, Watt SM, Murdoch SJ, Lister TA and Young BD (1991) Detection of additional JH/BCL2 translocations in follicular lymphoma. *Leukemia* **5**: 548–554

Ravetch JV, Siebenlist U, Korsmeyer S, Waldmann T and Leder P (1981) Structure of the human immunoglobulin locus: characterization of embryonic and rearranged J and D genes. *Cell* **27**: 583–591

Reed JC, Kitada S, Takayama S and Miyashita T (1994) Regulation of chemoresistance by the bcl-2 oncoprotein in non-Hodgkin's lymphoma and lymphocytic leukemia cell lines. *Ann Oncol* **5** (suppl 1): S61–S65

Richaud PM, Soubeyran P, Eghbali H, Chacon B, Marit G, Broustet A and Hœrni B (1998) Place of low-dose total body irradiation in the treatment of localized follicular non-Hodgkin's lymphoma: Results of a pilot study. *Int J Radiat Oncol Biol Phys* **40**: 387–390

Rohatiner AZ, Johnson PW, Price CG, Arnott SJ, Amess JA, Norton AJ, Dorey E, Adams K, Whelan JS, Matthews J, MacCallum PK, Oza AM and Lister TA (1994) Myeloablative therapy with autologous bone marrow transplantation as consolidation therapy for recurrent follicular lymphoma. *J Clin Oncol* **12**: 1177–1184

Sambrook J, Fritsch EF and Maniatis T (1989) *Molecular Cloning: A Laboratory Manual*. Cold Spring Harbor Laboratory Press, Cold Spring Harbor

Solal-Celigny P, Lepage E, Brousse N, Reyes F, Haioun C, Leporrier M, Peuchmaur M, Bosly A, Parlier Y, Brice P, Coiffier B and Gisselbrecht C (1993) Recombinant interferon alfa-2b combined with a regimen containing doxorubicin in patients with advanced follicular lymphoma. Groupe d'Etude des Lymphomes de l'Adulte. *N Engl J Med* **329**: 1608–1614

Soubeyran P, Eghbali H, Bonichon F, Coindre JM, Richaud P and Hœrni B (1988) Localized follicular lymphomas: prognosis and survival of stages I and II in a retrospective series of 103 patients. *Radiother Oncol* **13**: 91–98

Soubeyran P, Eghbali H, Bonichon F, Trojani M, Richaud P and Hœrni B (1991) Low-grade follicular lymphomas: analysis of prognosis in a series of 281 patients. *Eur J Cancer* **27**: 1606–1613

Soubeyran P, Cabanillas F and Lee MS (1993) Analysis of the expression of the hybrid gene bcl-2/IgH in follicular lymphomas. *Blood* **81**: 122–127

Tsujimoto Y, Croce CM (1986) Analysis of the structure, transcripts, and protein products ob bcl-2, the gene involved in human follicular lymphoma. *Proc Natl Acad Sci USA* **83**: 5214–5218

Vairo G, Innes KM and Adams JM (1996) Bcl-2 has a cell cycle inhibitory function separable from its enhancement of cell survival. *Oncogene* **13**: 1511–1519

Weiss LM, Warnke RA, Sklar J and Cleary ML (1987) Molecular analysis of the t(14;18) chromosomal translocation in malignant lymphomas. *N Engl J Med* **317**: 1185–1189

*© 1999 Cancer Research Campaign*
